# Fixation strength of biocomposite wedge interference screw in ACL reconstruction: effect of screw length and tunnel/screw ratio. A controlled laboratory study

**DOI:** 10.1186/1471-2474-11-139

**Published:** 2010-06-30

**Authors:** Antonio Herrera, Fernando Martínez, Daniel Iglesias, José Cegoñino, Elena Ibarz, Luis Gracia

**Affiliations:** 1 Department of Orthopaedic Surgery and Traumatology, Miguel Servet University Hospital, Zaragoza. Aragón Health Science Institute, Spain; 2 Department of Surgery, University of Zaragoza, Spain; 3 Department of Mechanical Engineering, University of Zaragoza, Spain

## Abstract

**Background:**

Primary stability of the graft is essential in anterior cruciate ligament surgery. An optimal method of fixation should be easy to insert and provide great resistance against pull-out forces.

A controlled laboratory study was designed to test the primary stability of ACL tendinous grafts in the tibial tunnel. The correlation between resistance to traction forces and the cross-section and length of the screw was studied.

**Methods:**

The tibial phase of ACL reconstruction was performed in forty porcine tibias using digital flexor tendons of the same animal. An 8 mm tunnel was drilled in each specimen and two looped tendons placed as graft. Specimens were divided in five groups according to the diameter and length of the screw used for fixation. Wedge interference screws were used. Longitudinal traction was applied to the graft with a Servohydraulic Fatigue System. Load and displacement were controlled and analyzed.

**Results:**

The mean loads to failure for each group were 295,44 N (Group 1; 9 × 23 screw), 564,05 N (Group 2; 9 × 28), 614,95 N (Group 3; 9 × 35), 651,14 N (Group 4; 10 × 28) and 664,99 (Group 5; 10 × 35). No slippage of the graft was observed in groups 3, 4 and 5. There were significant differences in the load to failure among groups (ANOVA/P < 0.001).

**Conclusions:**

Longer and wider interference screws provide better fixation in tibial ACL graft fixation. Short screws (23 mm) do not achieve optimal fixation and should be implanted only with special requirements.

## Background

Anterior cruciate ligament (ACL) reconstruction has become one of the most frequent procedures in arthroscopic surgery of the knee [1]. For many years, arthroscopic bone-tendon-bone reconstruction has been considered the gold standard operation in ACL surgery. In the last decade, the use of double looped hamstrings has been growing in popularity among arthroscopists. Excellent results have been reported with this technique [2,3], without significant differences between the outcomes of both procedures [4].

However, there is some degree of controversy about the increase of knee laxity during the first stages of the postoperative period after the use of hamstring tendons. Four bundle hamstrings grafts have more resistance than normal ACL and BTB grafts [5,6], but the primary fixation obtained by the use of an interference screw between bone blocks and bone tunnels is supposed to be better than fixation obtained with hamstrings and any other device.

Looking for a device that allows the use of hamstrings with a solid primary fixation has been the challenge for both, orthopaedic surgeons and bioengineers. This ideal device should be biocompatible, respectful with the graft and easy to insert. In addition, it should allow the placement as near as possible from the joint space to avoid/decrease the windshield-wiper effect and should be able to allow motion, weight-bearing and close kinetic chain exercises (CKCE) from the very early stages of rehabilitation [7]. During the last years, new methods of fixation have been developed on this basis.

Bio-absorbable implants would add the advantage of avoiding hardware permanence, intolerance and need of removal. In addition, these implants allow magnetic resonance explorations without misrepresentation of the obtained images [8].

However, there is some degree of discrepancy concerning the screw/tunnel ratio and implant length that should be recommended for an optimal primary fixation of the graft [9-12].

The purpose of this experimental study was to test the primary fixation obtained with a specific type of screw and to determine the effect of the screw/tunnel ratio and implant length in the resistance of the graft to traction forces.

The primary hypothesis of the study was: "*For a determined tibial tunnel diameter, longer and wider screws provide better fixation of the graft*"

## Methods

Forty rear limbs from young female or castrated male pigs were used in this study. Pigs were sacrificed at an industrial slaughterhouse after being stunned with CO_2_. Rear limbs were vacuum packed and stored refrigerated (4°C) for 24 hours and then manipulated at room temperature. Tibias and flexor digitorum profundus tendons were dissected from the rest of the tissue.

Tibias were placed in a holder and a 8 mm tunnel was performed with the Stryker^® ^ACL instrumentation. The direction of the tunnel was from the medial cortex of the proximal tibia to the lateral side of the anterior half of the medial tibial spine.

For each case, two flexor digitorum profundus tendons were selected and prepared to compose a double-looped graft with a cross-section optimal for the diameter of the tunnel (8 mm). The Stryker^® ^ACL Workstation was used for pre-tensing the graft and a 17 lbs (75,62 N) tension was applied.

The looped tendons were passed through the tunnel forming a four strand graft and then fixed with Biosteon^® ^Wedge interference screws (Stryker^®^) of different lengths and diameters. Screws were inserted until their rear end was at the level of the entrance of the tunnel (at the antero-medial cortex of the tibial shaft), a 1 mm nitinol guide wire was passed among the tendon bundles for this purpose. There was no protrusion of the tip of the screws at the articular surface of the tibia.

Five groups of testing were conformed, with 8 different specimens in each group. In all of them, the tunnel diameter was 8 mms but the screw selection was done according to the following distribution:

Group 1: Screws of 9 mm diameter and 23 mm length.

Group 2: Screws of 9 mm diameter and 28 mm length.

Group 3: Screws of 9 mm diameter and 35 mm length.

Group 4: Screws of 10 mm diameter and 28 mm length.

Group 5: Screws of 10 mm diameter and 35 mm length.

Tests were performed with a Servohydraulic Fatigue System (Universal Testing Machine Instron^® ^8800). A special fixture was designed which allowed accurate positioning of the tibia for applying longitudinal tension to the graft. A metal pin was passed through the loop formed by the graft and a second fixture was placed. Load and displacement were controlled and analyzed by the specific Instron^® ^software.

**Figure 1:**
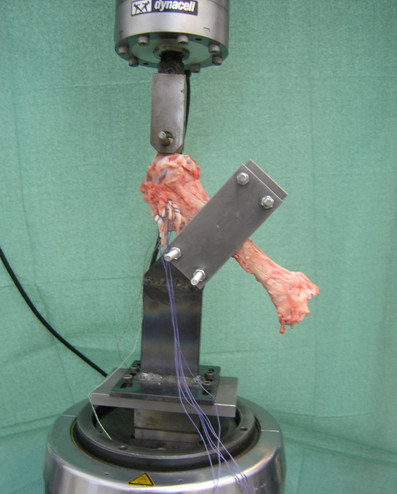
**Test arrangement**. Specimen adaptation to the Servohydraulic Fatigue System (Universal Testing Machine Instron^® ^8800) with a special fixture designed for this purpose.

Figure 1. Test arrangement

Specimens were peak loaded to failure at a rate of 2 mm/min. This speed causes a slow load increase, which according to ASTM (American Section of the International Association for Testing Materials) standards is appropriate for testing static friction, as it sets the lowest limit for pull-out resistance, since dynamic friction is higher.

Tests were finished when failure of the fixation (pull-out of the graft) or failure of the graft (elongation) were evident according to the resulting data (shown in real time in the computer monitor).

**Figure 2:**
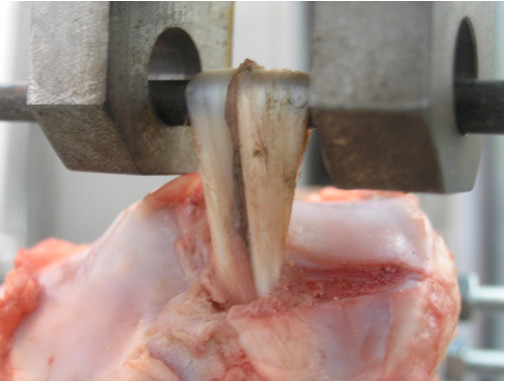
**Elongation of tendons without pull-out**. When no pull-out was observed, tendons suffer elongation as fibbers begin to fail.

Figure 2. Elongation of tendons without pull-out.

### Statistical evaluation

Descriptive statistical analysis was done with the determination of mean loads, standard deviation, median, minimum and maximum loads for each group. Confidence intervals (C.I.) for the mean value of the load at 95% were obtained applying the Student's t-test for a normal distribution of the sample.

Analysis of variance (ANOVA) was performed for testing the differences among the five groups. A post hoc pairwise comparison was done with the Fisher's protected least square difference test (PLSD). Differences with a significance level of P < 0.05 were considered as statistically significant.

## Results

**Figure 3:**
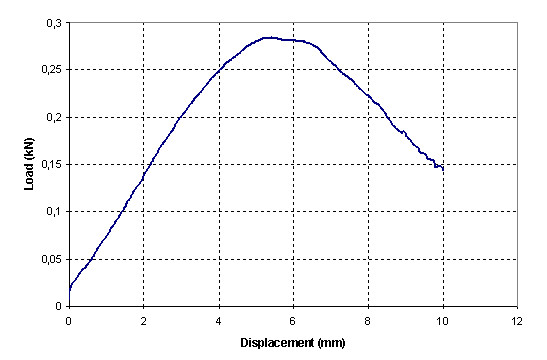
**Load-displacement curve for a test with pull-out**. Sudden load decrease when the graft bundles begin to slide through the tunnel, the load decreases continuously as the sliding progresses.

**Figure 4:**
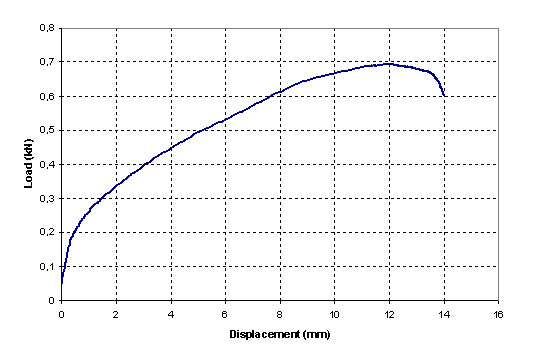
**Load-displacement curve for a test with elongation of tendons**. Smooth load decrease when stretching of the graft begins, with rough and sudden load fall when the first breaking happens.

The load/displacement type curve for the tests where fixation failure was observed is shown in Figure 3. In this curve there is an evident load decrease at the moment when the graft bundles begin to slide through the tunnel, the load decreases continuously as the sliding progresses. In the case of graft failure due to fatigue of the tendon fibers, the final of the load/displacement curve is different (Figure 4); load smoothly decreases when stretching of the graft begins and a rough and sudden load fall is observed when the first breaking happens.

Relevant results for each group are shown in Table 1.

**Table 1:** Test results

Group	Cases	Tunnel Diam.	Screw Diam	Screw Length	Mean Load	St. Dev.	Type of Failure	Confidence Int. (95%)
1	8	8 mm	9 mm	23 mm	295,44 N	10,16 N	Fixation- (8) Graft- (0)	286.94 303.93
2	8	8 mm	9 mm	28 mm	564,05 N	75,64 N	Fixation- (4) Graft- (4)	496.45, 631.65
3	8	8 mm	9 mm	35 mm	614,95 N	14,31 N	Fixation- (0) Graft- (8)	602.16 627.74
4	8	8 mm	10 mm	28 mm	651,14 N	35,89 N	Fixation- (0) Graft- (8)	619.06 683.22
5	8	8 mm	10 mm	35 mm	664,99 N	38,20 N	Fixation- (0) Graft- (8)	630.84 699.13

Characteristics of the groups, Obtained Mean Load with Standard Deviation, Type of Failure and Confidence Intervals for the mean value of the load at 95% (Student's t-test/normal distribution of the sample).

In Group 1 there was evident fixation failure (graft pull-out) of all the specimens. when a load of 295.4 N +/- 7.92 N was reached. C.I. (286.94, 303.93).

In Group 2, the born load was 565.3 N +/- 90.93N, with 4 cases of evident fixation failure, and 4 cases of graft failure. C.I. (496.45, 631.65).

In Group 3 there wasn't any evident fixation failure (late slippage in 3 cases, but with loads higher than 600 N and after elongation of the graft was evident). The obtained load was 617.45 N +/- 11.99. C.I. (602.16, 627.74).

In Group 4 there wasn't any cases of fixation failure, with graft failure in all the specimens. The reached load was 650.13 N +/- 22.8 N. C.I. (619.06, 683.22).

In Group 5 there weren't any cases of fixation failure, with elongation of the graft in all the specimens. The reached load was 665.6 N +/- 38.47N. C.I. (630.84, 699.13).

These differences in the load to failure among the groups were significant (ANOVA/p < 0.0001). The pairwise comparison (Fisher's PLSD) showed that, when comparing group to group, differences in the mean load to failure were significant except when comparing group 3 with group 4 and group 4 with group 5.

**Figure 5:**
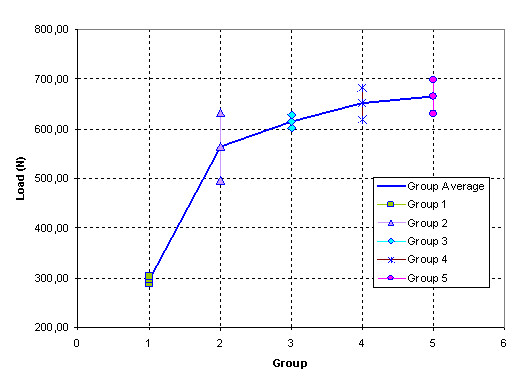
**Load evolution for every test group**. Mean load and range of values for each group. Loads increase with the length and diameter of the screw.

Figure 5 shows the evolution of the load depending on the tested group with the confidence bounds.

## Discussion

In ACL surgery, primary stability of the graft is essential for allowing early rehabilitation during the post-operative period and a solid fixation of the graft in the tibial tunnel is required for this purpose [13-15]. In this study, experimental testing of a screw tibial fixation for hamstrings tendons has been performed. This device is designed for four-strand grafts; this surgical technique has being gaining ground on bone-tendon-bone procedures because of its low morbidity and good results [2-4], although two-bundle grafts have demonstrated to achieve enough stability [16]. Bio-absorbable screws have evident advantages [8]: they provide similar or even better fixation than metallic screws [17-20], causing less damage to the graft [21] and without interfering with the graft incorporation process [19]. Zantop et al [22] reported less laceration of the graft with poly-D,L-lactide (PLDLA) screws than with titanium screws, the results were better when a composite of PLDLA and Tricalcium phosphate was used.

Post-operative stability of the graft is essential during the first stages, after four weeks collagen Sharpey-like fibers begin to develop in the bone-graft interface [23] and biological fixation will be established. Synthesis of these fibers from postoperative formation of granulation tissue is easier in tendinous grafts than in grafts including bone blocks [19]. Thus, key for success and knee stability depends on the capability of the fixation device to allow weight bearing and early mobilization of the knee.

An optimal study design should include human young fresh cadaveric tibias, but this kind of specimens are difficult to obtain and are destined to be used as allograft. The use of cadaveric bones from elderly specimens is related with low bone mineral density (BMD) and poor fixation strength [24-26]. For these reasons, the use of an animal model is an obvious solution for a controlled laboratory study. Porcine knee models have demonstrated to be a good alternative to cadaveric bone [27-29]; whereas bovine models are correlated with higher traction resistance than human [29-31] or porcine [28,30-33] models. In the tests performed by Nagarkatti et al. [34], the average density of porcine bone was similar to that of young human bone, and significantly higher than that of elderly human cadaveric bone.

**Figure 6:**
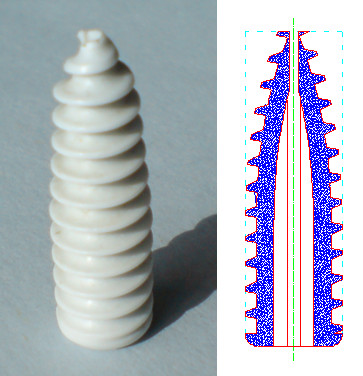
**Geometry of Biosteon^® ^screw**. A wedge tip cannulated screw. Distal diameter is smaller providing less contact with the graft and the tunnel. Inverted threads apply traction to the graft as the screw advances through the tunnel.

Biosteon^® ^is a biocomposite made out of Poly L-Lactic Acid and Hydroxyapatite which increases the biocompatibility of the implant and provides osteoconductive potential [35]. This wedge-tip interference screw design (Figure 6) facilitates its insertion even with screws of diameters 1 or 2 mm larger than the tunnel diameter. On the other hand, the wedge tip design makes the diameter of the distal third of the screw smaller than the diameter of rest of the screw. There is less contact between the screw and the bone walls in the proximal portion of the tibial tunnel. During the experimental testing it was obvious that the shortest screws (23 mm length) were the ones that obtained the worst pull-out values (slightly less than 300 N). This difference was significant (p < 0,0001) when comparing group 1 with any of the other groups (Table 2). These values wouldn't guarantee proper fixation during the postoperative usual exercises [36,37].

**Table 2:** Statistical Analysis

Compared Groups		Compared Screws		Mean Difference	Critical Difference	P- Value	Statistical Significance
1	2	9 × 23	9 × 28	268,613 N	45,466 N	< 0,0001	Yes
1	3	9 × 23	9 × 35	319,512 N	45,466 N	< 0,0001	Yes
1	4	9 × 23	10 × 28	355,700 N	45,466 N	< 0,0001	Yes
1	5	9 × 23	10 × 35	369,550 N	45,466 N	< 0,0001	Yes
2	3	9 × 28	9 × 35	50,900 N	45,466 N	0,0293	Yes
2	4	9 × 28	10 × 28	87,087 N	45,466 N	0,0004	Yes
2	5	9 × 28	10 × 35	100,938 N	45,466 N	< 0,0001	Yes
*3*	*4*	*9 × 35*	*10 × 28*	*36,188*	*45,466 N*	*0,1151*	*No*
3	5	9 × 35	10 × 35	50,038	45,466 N	0,0320	Yes
*4*	*5*	*10 × 28*	*10 × 35*	*13,850*	*45,466 N*	*0,5403*	*No*

Post hoc pairwise comparison with the Fisher's protected least square difference test (PLSD).

With 28 mm length screws and diameter 1 mm wider than the tunnel (9 mm) there was a mean bore load of 564,05 N. When increasing the length to the screw to 35 mm a significant increase (p = 0,0293) in the mean peak load was found. Even better results were obtained using screws with diameter 2 mm wider than the tunnel, with no significant differences between 28 and 35 mm implants.

These results are slightly lower than those reported by Kousa el al. [18] in a similar porcine study, but in their paper there were two main differences: they used a bone tendon-bone type graft, and they performed their peak load tests at a rate of 50 mm/min; which measures mainly dynamic friction. In our tests, the rate of 2 mm/min determined the static friction, which is the lowest limit for pull-out resistance. Main differences in the design of biomechanical studies (bovine, porcine or human specimens; bone blocks or tendon fixation, metal or absorbable screws) make difficult to compare data obtained in different studies.

These loads are higher than the ones reported during the activities of the initial stages of rehabilitation after ACL surgery like isokinetic/isometric extension of the knee [37] or quadriceps muscle pull against gravity [36]; in fact, the graft is loaded less than 500 N during daily living activities [6,38,39].

These results confirm the importance of the length of the interference screw. With longer screws it is easier to place the insertion torque near of the joint line [11,13,40]. Some studies, however, showed little effect of the screw length [9,11]. The effect of the screw diameter is another point of discrepancy. In this experimental study, there was an evident improvement with wider screws, which correlates with some other studies [41].

Morris et al. [10], in a porcine model, observed serious damage in the graft due to cut-out with wider screws. In our testing, we found no differences and there were no evident damage to the graft with any of the screws, perhaps because the shape of the screw is crucial in the insertion and facilitates the implantation of wider screws without graft damage. Weiler et al. [12] in a bovine model concluded that increasing the length of the screw improved tibial fixation more than oversizing the diameter of the screw, but this study was done with 23 and 28 mm screws. In our model, for screws longer than 23 mm, the improvement was more evident when oversizing the screw (Table 1: Group 2 to Group 4 = + 87.09 N) than when using a longer screw (Table 1: Group 2 to Group 3 = + 50.90 N).

As Buelow et al reported [42] the insertion of an interference screw not only compresses the graft in the tunnel but also leads to an enlargement of the tunnel itself. The use of wider screws determine the presence of wider tibial tunnels in the postoperative period (in X-rays, CT or MRI) but with no further enlargement and without any effect in the outcome of the procedure.

According to this experimental testing, the 23 mm screw should only be used in exceptional situations when there is a very short tibial tunnel. With 28 mm screws and an oversized diameter of +1 mm, traction forces higher than 500 N are necessary to obtain some degree of slippage of the graft. With an increase in the length or the diameter of the screw there isn't any kind of slippage, even with loads higher than 600 N. These results are similar or better than the ones obtained in previous studies [10,12,41,43-45].

With this type of screw (Biosteon^® ^Wedge Interference Screw) and a minimal length of 28 mm, primary stability of the graft is achieved and early mobilization of the knee can be allowed. The resistance to traction forces achieved is 200 N higher than the biomechanical requirements for walking with monopodal weight bearing [44] and could be enough for jumping. The use of 2 mm oversized screws and the maximal length of the implant is recommended to increase primary fixation.

## Conclusions

1. Screws 1 mm wider than the tunnel with a minimal of 28 mm length resist loads higher than 500 N.

2. Longer and wider interference screws provide better fixation in tibial ACL graft fixation.

3. Short screws (23 mm) do not achieve optimal fixation and should be implanted only under special requirements.

## Competing interests

The authors declare that they have no competing interests.

## Authors' contributions

AH conceived the design of this study, coordinated the work between surgeons and engineers and participated in the acquisition of data and in the drawing up of the manuscript. FM conceived the design of the study, performed the surgical preparation of the specimens and participated in the acquisition of data and in the drawing up of the manuscript. DI assisted during the surgical process and participated in the acquisition of data. JC prepared and adapted the hardware for testing the specimens, performed the fatigue tests, participated in the acquisition of data and in the interpretation of the resulting curves. EI assisted during the mechanical testing and participated in the acquisition of data. LG conceived the design of this study, coordinated the work between surgeons and engineers and participated in the acquisition of data and in the drawing up of the manuscript. All authors read and approved the final manuscript.

## Pre-publication history

The pre-publication history for this paper can be accessed here:

http://www.biomedcentral.com/1471-2474/11/139/prepub
